# Glioblastoma Multiforme Selective Nanomedicines for Improved Anti-Cancer Treatments

**DOI:** 10.3390/pharmaceutics14071450

**Published:** 2022-07-12

**Authors:** Jason Thomas Duskey, Arianna Rinaldi, Ilaria Ottonelli, Riccardo Caraffi, Chiara Alessia De Benedictis, Ann Katrin Sauer, Giovanni Tosi, Maria Angela Vandelli, Barbara Ruozi, Andreas Martin Grabrucker

**Affiliations:** 1 Nanotech Lab, Te.Far.T.I., Department of Life Sciences, University of Modena and Reggio Emilia, 41125 Modena, Italy; jasonthomas.duskey@unimore.it (J.T.D.); arianna.rinaldi@unimore.it (A.R.); ilaria.ottonelli@unimore.it (I.O.); riccardo.caraffi@unimore.it (R.C.); gtosi@unimore.it (G.T.); mariaangela.vandelli@unimore.it (M.A.V.); 2 Clinical and Experimental Medicine PhD Program, University of Modena and Reggio Emilia, 41125 Modena, Italy; 3 Department of Biological Sciences, University of Limerick, V94 T9PX Limerick, Ireland; chiara.debenedictis@ul.ie (C.A.D.B.); ann.katrin.sauer@ul.ie (A.K.S.); 4 Bernal Institute, University of Limerick, V94 T9PX Limerick, Ireland; 5 Health Research Institute (HRI), University of Limerick, V94 T9PX Limerick, Ireland

**Keywords:** drug delivery, drug targeting, NMeds, glioblastoma, brain tumour, nanomedicine, cancer, nanoparticles, improved chemotherapeutics

## Abstract

Glioblastoma Multiforme (GBM) is a devastating disease with a low survival rate and few efficacious treatment options. The fast growth, late diagnostics, and off-target toxicity of currently used drugs represent major barriers that need to be overcome to provide a viable cure. Nanomedicines (NMeds) offer a way to overcome these pitfalls by protecting and loading drugs, increasing blood half-life, and being targetable with specific ligands on their surface. In this study, the FDA-approved polymer poly (lactic-co-glycolic) acid was used to optimise NMeds that were surface modified with a series of potential GBM-specific ligands. The NMeds were fully characterised for their physical and chemical properties, and then in vitro testing was performed to evaluate cell uptake and GBM cell specificity. While all targeted NMeds showed improved uptake, only those decorated with the-cell surface vimentin antibody M08 showed specificity for GBM over healthy cells. Finally, the most promising targeted NMed candidate was loaded with the well-known chemotherapeutic, paclitaxel, to confirm targeting and therapeutic effects in C6 GBM cells. These results demonstrate the importance of using well-optimised NMeds targeted with novel ligands to advance delivery and pharmaceutical effects against diseased cells while minimising the risk for nearby healthy cells.

## 1. Introduction

Glioblastoma Multiforme (GBM) is an incredibly devastating disease that is immediately considered a grade 4 cancer diagnosis due to its very high motility and growth rate [1,2,3,4]. GBM affects approximately 17,000 people annually, creating vast amounts of healthcare and treatment costs for patients and their families that have been estimated at up to $250,000 per patient [5,6]. After diagnosis, currently accepted treatments include surgical tumour resection followed by radiotherapy and concomitant chemotherapy with temozolomide (TMZ) [7,8]. Unfortunately, studies show that even with this combined approach, fewer than 25% of patients survive more than two years [9,10]. The major limitations of these treatments lie in the significant side effects of the chemotherapeutics and the inability of those drugs to selectively eliminate residual GBM cells, which often leads to recurrences. In this view, nanomedicine (NMed) delivery systems offer crucial advantages. NMeds can be formulated to improve the solubility, biodistribution, and bioavailability of previously incompatible chemotherapeutics [11,12,13,14]. Moreover, targeted NMeds hold a considerable advantage over other traditional delivery methods. A rapidly increasing number of studies are being published searching for novel ligands to be incorporated onto the surface of NMeds to achieve specific delivery to organs [15,16,17,18], cells [19,20], or even intracellular locations [21,22,23], with several studies focusing on permeating the blood–brain barrier (BBB) for brain targeting [24,25,26,27,28,29]. When designing NMeds for GBM targeting, there are several barriers to overcome. For a ligand to be GBM specific, it should not only be able to target GBM cells, but also to deliver the cargo across the BBB. At the same time, delivering toxic anticancer drugs to the brain often leads to severe toxicity and can drastically affect the healthy cells (astrocytes, neurons, etc.) and disrupt proper brain functions [30,31,32,33,34,35]. Thus, identifying a ligand that is non-saturable, capable of transporting nano-sized cargo, and has high affinity and specificity for GBM could significantly increase the therapeutic potential and lower the off-target toxicity of NMeds loaded with chemotherapeutic agents.

Based on the most recent literature, several ligands that have shown potential for brain targeting can also be evaluated regarding their tropism for specific cell populations such as brain cancer and GBM [36,37,38,39]. This work aimed to evaluate the targeting potential of different surface-decorated NMeds to determine their specificity and ability to deliver nano-sized cargo to GBM cells. Thus, four ligands were tested: two peptides, g7 and AAVF, and two monoclonal antibodies, M08J and M08. The peptides g7 and AAVF are already published for their BBB crossing potential. The g7 peptide (sequence GFtGFLS[O-ß-D-Glucose]) is an opioid receptor ligand that, when attached to the surface of NMed, has been demonstrated to cross the BBB at up to 10% of the injected dose and improve the brain delivery of a variety of therapeutic molecules [16,36,40,41,42]. The peptide AAVF (sequence FVVGQSY) is a short peptide from the adeno-associated virus protein coat, which was found through phage display studies and has recently been shown to have potential BBB targeting effects [43,44,45,46]. While this is already a huge bonus for the delivery of pharmaceutics across the BBB, these ligands have been demonstrated to have the potential for GBM specificity as well, but more thorough evaluations are required [47,48,49,50]. The two antibodies, M08J and M08, are two commercially available isoforms of Cell Surface Vimentin (CSV) antibodies that have different activities due to non-disclosed proprietary reasons [51]. CSV is an intermediate filament protein that is naturally expressed in numerous cell types such as lymphocytes, macrophages, and fibroblasts etc., and it was found to be important in epithelial–mesenchymal transition (EMT) [52,53]. Further evidence has also linked CSV to tumour growth, evidenced by its upregulation in various cancer types ranging from oral cancer, breast cancer, colon cancer, prostate cancer etc [53,54,55,56,57]. The presence of CSV in brain cancer and model cell lines has also been shown for numerous tumours in the CNS; however, the amounts of CSV are highly dependent on cell type, patient, and also treatment regimens [58,59,60,61]. While CSV is often upregulated in GBM, it has also been shown to be expressed at high levels in the brain endothelium cells, suggesting that these ligands could help in both BBB crossing and specifically targeting GBM cells [61,62,63,64].

To evaluate their targeting potential, polymeric NMeds composed of the biodegradable and biocompatible polymer poly(D,L-lactic-co-glycolic) acid (PLGA) were optimised and fully characterised for improved surface modification with the ligands. The formulation and surface modification protocols were further optimised to reduce reagent loss and make the formulation and modification more “green”. Optimised surface decorated NMeds were then tested in vitro to assess their cell uptake in GBM (C6) and healthy astrocyte (DI TNC1) cell cultures, as well as their effect on cell viability. Co-culture experiments further demonstrated the targeting capacity of these ligand-targeted NMeds and their effects on cell growth. Finally, the most promising targeted NMed candidate was loaded with the anticancer drug paclitaxel (PTX) to evaluate its anti-cancer effect on GBM cells. These results revealed the potential of NMeds with the novel ligands to enhance transport, cell uptake, and specificity to GBM cells. This improvement could greatly increase the chances of creating a functional therapeutic that minimises damage to the nearby cells in the central nervous system (CNS) while increasing chemotherapeutic effectiveness.

## 2. Materials and Methods

### 2.1. Materials

Resomer^®^ RG 503H Poly(D,L-lactide-co-glycolic) acid 50:50 (PLGA) MW 11,000–12,000 was purchased from Evonik (Essen, Germany). 4-Morpholineethanesulfonic acid (MES, MW 195.24), N-(3-Dimethylaminopropyl)-N′-ethylcarbodiimide (EDC, MW 155.24), N-Hydroxysuccinimide (NHS, MW 115.09); Pluronic^®^ F68, D-(+)-Trehalose dihydrate (MW 378.33), acetone, acetonitrile (ACN), barium chloride (BaCl_2_), and iodine (I_2_) were purchased from Sigma-Aldrich (Milan, Italy). 1-[6-(6-aminohexylamino)-6-oxohexyl]-3,3-dimethyl-2-[(1E,3E,5E)-5-(1,3,3-trimethylindolin-2-ylidene)penta-1,3-dienyl]-3H-indolium chloride hydrochloride (Cy5 amine, MW 653.77) was purchased from Lumiprobe (Hannover, Germany). PAAVF peptide (MW 943.47) was purchased from GenScript (Piscataway, NJ, USA). The g7 peptide (MW 888.97) was purchased from Mimotopes Pty Ltd. (Mulgrave, VA, Australia). Cell Surface Vimentin (CSV) monoclonal antibody, clone 84-1, H00007431-M08 (M08), and Cell Surface Vimentin (CSV) monoclonal antibody, clone 84-1, H00007431-M08J (M08J), were purchased from Abnova (Taipei, Taiwan). Dichloromethane (DCM), trifluoroacetic acid (TFA), and potassium iodide (KI) were purchased from Carlo Erba (Milan, Italy). Hydrochloric acid 36% (HCl) was purchased from Avantor (Radnor Township, PA, USA). Paclitaxel (PTX, MW 853.906, CAS 33069-62-4) was purchased from Selleckchem (Houston, TX, USA). MilliQ water was purified by a Millipore system (Millipore, Bedford, MA, USA). All chemicals used were of analytical grade. If not otherwise mentioned, all other chemicals and reagents were purchased from Sigma Aldrich (Milan, Italy).

### 2.2. NMed Formulation

#### 2.2.1. Synthesis of PLGA-Cy5

PLGA was conjugated to Cyanine 5 (Cy5) via an amide bond formation using an already optimised protocol [65,66]. Briefly, PLGA (1 g, 88 μmol) was solubilised in anhydrous dioxane (15 mL) under magnetic stirring at 10 °C. The PLGA was activated by added NHS (12 mg) and DCC (22 mg) and left to react at room temperature for 4 h. The by-product, dicyclohexylurea, was removed by paper filtration, and cyanine 5 amine (43 mg) was added to the purified activated polymer. The pH was adjusted to 7–8 with TEA, and the reaction was allowed to continue for 7 h at room temperature. Next, the conjugated polymer was purified by precipitation in ether. The basic pH was neutralised by the addition of ether saturated with HCl. Finally, the polymer was precipitated in methanol overnight, followed by several steps of centrifugation at 10,000 rpm for 10 min (ALC multispeed centrifuge PK 121) to separate the product. The PLGA-Cy5 product was stored at −20 °C for future use.

#### 2.2.2. Optimisation of the NMed Formulations

PLGA NMeds were formulated via the nanoprecipitation technique. First, PLGA and the PLGA-Cy5 conjugate were solubilised in 4 mL of acetone and vortexed (total polymer weight = 50 mg) (Advanced Vortex Mixer ZX3, Velp^®^ Scientifica). This solution was added dropwise into a beaker containing 12.5 mL of Pluronic^®^ F68 under magnetic stirring (Multistirrer, Magnetic Stirrer Velp^®^ Scientifica, Usmate Velate, Italy) for 2 h at room temperature. The NMed suspension was purified by centrifugation at 9700 rpm for 10 min and resuspended in 4 mL of Pluronic^®^ F68 1.5% *w*/*v*. The NMed suspensions were stored at 4 °C for further analysis.

This general method was used to optimise the amount of PLGA-Cy5, ranging from 0.1–4% *w*/*w* of total PLGA. Further optimisations were then performed by maintaining the amount of PLGA-Cy5 constant at 0.2% *w*/*w* while varying the amount of Pluronic^®^ F68 concentration in the aqueous phase (12.5 mL) from 0–3% *w*/*v*. Finally, as previously indicated, the purified by centrifugation and the NMeds were resuspended in 4 mL of 1.5% *w*/*v* Pluronic^®^ F68.

### 2.3. Optimisation of the Post-Modification Surface Modification Reaction

Reaction of the ligands to the NMed surface was performed in 0.1 M MES at pH 4.9. To achieve this composition, NMeds were initially centrifuged after formulation and resuspended in MES solution 0.1 M; however, this method required two centrifugation steps which hampered the NMed stability and led to aggregation. To overcome this, the first centrifugation was avoided by adding 2 mL of MES solution 5× (0.5 M) directly to the NMed suspension (10 mL, approx. 4 mg/mL) to achieve a final concentration of 100 mM pH 4.9. To the buffered NMed suspension, 300 mg of EDC were added and left to react for 10 min, followed by the addition of 100 mg of NHS. This reaction was left stirring at room temperature for 20 min, and the activated NMeds were characterised for their physicochemical characteristics, weight yield %, and % residual surfactant.

To complete the post-modification reaction, 1 or 10 µg of each ligand, g7, AAVF, M08J, or M08, were added to the activated NMed suspension (10 µg/40 mg NMeds). The reaction was maintained stirring for 1.5 h at room temperature. The suspension of surface-modified NMeds was purified by centrifugation for 10 min at 9700 rpm to form a pellet. The supernatant was discarded, and the pellet was resuspended in 4 mL of Pluronic^®^ F68 1.5% *w*/*v* and stored at 4–8 °C until used.

### 2.4. NMed Characterisation

#### 2.4.1. Size and Zeta Potential Analysis

The particle size, Polydispersity Index (PDI), and Z-potential were measured by diluting 10 μL of the purified NMeds in 1 mL of MilliQ water (final concentration of 0.01 mg/mL) and analysed using Photon Correlation Spectroscopy (PCS): Laser 4 mW He–Ne, 633 nm, Laser attenuator Automatic, transmission 100–0.0003%, Detector Avalanche photodiode, Q.E. > 50% at 633 nm, T = 25 °C (Zetasizer Nano ZS, Malvern, Malvern, UK). All samples were analysed in triplicate of at least three independent NMed formulations.

#### 2.4.2. Microscopy Analysis by AFM

The morphology of the targeted NMeds was evaluated by Atomic Force Microscopy (AFM, Park Instruments, Sunnyvale, CA, USA) at RT operating in air and non-contact mode using triangular silicon tips. The resonant frequencies of the cantilever were found to be in the range of 160 kHz. Before the analysis, the NMeds were diluted to 0.01 mg/mL, applied to a small mica disk (1 cm × 1 cm), and analysed after removing the excess solution. The topographical images were flattened using second-order fitting to remove sample tilt.

#### 2.4.3. Weight Yield

Purified NMed aliquots of 0.5 mL were lyophilised (LyoLab 3000, Heto-Holten, ThermoFisher Scientific, Waltham, MA, USA) in pre-weighed Eppendorf tubes, and the weight yield % was calculated as follows:

yield % = ((mg product − mg Pluronic^®^ F68 used in resuspension)/mg total PLGA) × 100

#### 2.4.4. Pluronic^®^ F68 Quantification

The residual amount of Pluronic^®^ F68 remaining in the formulated NMeds was evaluated using a previously published colorimetric method [67]. NMeds were solubilised in 0.5 mL of DCM and then slowly added to 10 mL of water. The organic solvent was evaporated by stirring at room temperature to precipitate the PLGA, which was then removed by filtration (cellulose nitrate filter, porosity 0.45 m, Sartorius, Firenze, Italy). An amount of 2 mL of the aqueous solution was treated with 2 mL of 0.5% *w*/*v* BaCl_2_ in HCl 1 N and 0.5 mL of I_2_/KI (0.05 M/0.15 M). This mixture was incubated for 10 min in the dark. Then, the Pluronic^®^ F68 concentration was calculated using a spectrophotometer (Model V530, Jasco, Cremella, Italy) measuring the absorbance at 540 nm, using a calibration curve made from stock solutions of Pluronic^®^ F68 prepared under the same experimental conditions. Linearity was found in the range of 4–48 µg/mL of Pluronic^®^ F68 (R^2^ = 0.9927). Due to the sensitivity of the I_2_/KI aqueous solution to heat and light, the standard curve was calculated fresh each day. The analysis was performed in triplicate on three different NMed formulations. The residual Pluronic^®^ F68 was calculated as follows:

% residual Pluronic^®^ F68 = (quantified mg Pluronic^®^ F68/mg NMeds) × 100

#### 2.4.5. Storage Stability

NMed suspensions were aliquoted (50 µL), stored at 4 °C, frozen at −20 °C, or lyophilised over 3 weeks. Frozen and lyophilised samples were supplemented with the cryoprotectant trehalose at ratios ranging from 0.1–3 *w*/*w* of the NMeds. After 3 weeks, the samples were thawed, brought to room temperature, and analysed for size, PDI, and Z-potential.

#### 2.4.6. Paclitaxel NMed Formulations

Paclitaxel (PTX) loaded PLGA NMeds were formulated using a modified protocol from the previous nanoprecipitation (without the presence of PLGA-Cy5). First, PTX (0.5 mg) was solubilised in 1 mL of acetone and added to 50 mg of PLGA solubilised in 3 mL of acetone to arrive at 4 mL. The solution was vortexed (Advanced Vortex Mixer ZX3, Velp^®^ Scientifica) and added dropwise into a beaker containing 12.5 mL of Pluronic^®^ F68 1.5% *w*/*v*, and the acetone was evaporated by magnetic stirring (Multistirrer, Magnetic Stirrer Velp^®^ Scientifica) for 2 h at RT. Following formation, the NMeds were either modified with M08 as previously described using the post-modification method or subjected to a mock post-modification reaction without M08 in the solution (non-modified). The NMeds were purified by centrifugation at 9700 rpm for 10 min, resuspended in 4 mL of Pluronic^®^ F68 1.5% *w*/*v*, and analysed for size, PDI, and Z-potential as previously described.

The amount of PTX in the NMeds was quantified using HPLC analysis (Jasco Europe, Cremella, Italy): the system consisted of a PU-2089 Pump with a 50 µL sample loop (model 775i). The column used for analysis was a 5HC-C18 250 × 4.6 mm (Agilent, Cernusco sul Naviglio, Italy). The mobile phases consisted of: (A) H_2_O + 0.1% (*v*/*v*) TFA and (B) ACN. The optimised gradient consisted of increasing phase B from 50–90% ACN over 10 min which yielded to a PTX retention time of 7 min. The absorbance was monitored at 210 nm using a UV detector (Jasco UV-1575). NMeds (~2 mg) were lyophilised, and the paclitaxel was liberated from the NMeds by adding 0.5 mL DCM and 0.5 mL of ACN. The solution was then magnetically stirred until all the DCM evaporated, precipitating the PLGA. ACN was then added to arrive at a final volume of 1 mL, and 50 µL were injected into the HPLC. The encapsulation efficiency of PTX (% EE) and loading content (% LC) were calculated using a standard curve calculated with pure PTX in ACN. The following formulas were used:

% EE = (measured PTX/feeding PTX) × 100

% LC = (measured PTX/amount of NMeds analysed) × 100

### 2.5. In Vitro Studies

#### 2.5.1. Cell Culture

C6 Rat GBM cells (ATCC/LGC Standards, Teddington, UK) were cultured in Ham’s F-12K medium with 20% FBS and 1% penicillin/streptomycin (Sigma Aldrich/Merck Life Science, Carrigtwohill, Ireland). DI TNC1 Rat Astrocyte cells (ATCC/LGC Standards, UK) were grown in DMEM High Glucose medium with 10% FBS and 1% penicillin/streptomycin. All cell lines were kept at 37 °C and 5% CO_2_.

#### 2.5.2. NMed Uptake Studies in C6 Cells

C6 cells were seeded at a density of 30,000 cells on PLL-coated glass coverslips inserted in a 24 well plate and grown overnight. Calcein-AM dye was prepared in a stock solution of 33.51 mM in DMSO and used at a final working concentration of 3 µM in an F12-K medium. The cells were incubated with Calcein-AM for 30 min at 37 °C, after which the staining solution was removed and replaced with fresh media.

Control (non-targeted) and targeted NMeds (with 1 µg or 10 µg of ligand) were added to the cell media at the final concentrations of 25, 50, or 100 µg/mL. After either 3 or 24 h, the media was removed, and cells were fixed with 4% PFA. Next, cells were washed 3× with 1× PBS, and the cell nuclei were stained with DAPI. The coverslips were mounted using Vecta Mount (Vector Laboratories, (2BScientific), Oxfordshire, UK). The cells were imaged using a Molecular Devices Imagexpress high content imaging confocal microscope, and image analysis was performed using ImageJ (v. 1.52q). Cell health was assessed by measuring the number of DAPI-stained nuclei per optic field of view, during which bright and condensed nuclei indicating cell death were excluded. NMed uptake was measured by quantifying intracellular Cy5 fluorescence intensity normalised for background fluorescence detected in untreated cells.

#### 2.5.3. NMed Uptake Study in Co-Culture (C6 Glioblastoma/DI TNC1 Astrocytes)

C6 cells were grown in t12 flasks. For the Calcein-AM staining, the old F12K medium was removed, the flask was washed twice with 1× PBS, and F12K supplement/serum-free medium (2 mL) was added to the cells containing the dye (final concentration 2 µM). The cells were incubated at 37 °C for 30 min, after which, the medium with the dye was removed, the cells were washed twice with 1× PBS, and fresh F12K complete medium (+supplements and FBS) was added. Subsequently, the cells were detached with Trypsin/EDTA and the C6 cells were seeded at low density in a 96 multi-well plate (2000 cells/well, 100 µL per well). The DI TNC1 Astrocytes were grown in t12 flasks in parallel, detached with Trypsin/EDTA, and seeded at 4000 cells/well (adding 100 µL per well). The co-cultures were grown overnight, and then the medium was removed from each well and replaced with 100 µL of complete F12K and 100 µL of complete DMEM. The NMeds were added to the co-culture at concentrations of 50 µg/mL and 100 µg/mL (either control NMeds (non-targeted) or each of the targeted NMeds (at 1 µg or 10 µg ligand in the post-modification reaction)). The co-culture was incubated for 3 h at 37 °C, the media was removed, and the cells were fixed with 4% PFA. Following fixation, the cells were washed 3× with 1× PBS, and the cell nuclei were stained with DAPI. Coverslips were mounted using Vecta Mount (Vector Laboratories, Burlingame, CA, USA). Cells were imaged using a Molecular Devices Imagexpress high content imaging confocal microscope, and image analysis was performed using ImageJ (v. 1.52q). Cell health was assessed by measuring the number of all DAPI positive nuclei, and DAPI positive nuclei in Calcein-AM positive cells per optic field of view in which bright and condensed nuclei indicating cell death were excluded. NMed uptake was measured by quantifying intracellular Cy5 fluorescence intensity normalised for background fluorescence detected in untreated cells.

#### 2.5.4. PXT NMed Toxicity Analysis

C6 cells were seeded at a density of 5000 cells/well on a PLL-coated E-Plate VIEW 16 plate (ACEA Biosciences, San Diego, CA, USA). The C6 cells were grown for 12.5 h before treatment with NMeds delivering the equivalent of 500 nM PTX and 500 nM free PTX and then monitored for another 14 h. Cell impedance was measured every 15 min using an ACEA xCELLigence RCTA DP system, where a decrease in impedance is associated with the detachment of cells and, therefore, a sign of cell death.

#### 2.5.5. PXT NMed Apoptosis Assay

Healthy and apoptotic cells were measured using a healthy/apoptotic/necrotic cell detection kit (Promokine) according to the manufacturer’s protocol. In brief, 30,000 C6 cells were seeded on PLL-coated glass coverslips inserted in a 24 well plate and grown overnight. NMeds were added to the cell media at concentrations to deliver the equivalent of 10, 50, 100, and 500 nM PTX. After 3 h of incubation, the medium was removed, cells were washed with 1× binding buffer, and incubated for 15 min at RT protected from light with a staining solution containing 100 μL of 1× binding buffer, 5 μL of FITC-Annexin V, and 5 μL of Hoechst 33342. Then, the cells were washed twice with 1× binding buffer and fixed in a solution containing 4% PFA in 1× PBS and 1.25 mM CaCl_2_ at RT for 15 min. Coverslips were mounted using Vecta Mount (Vector Laboratories, Burlingame, CA, USA), and the cells were imaged using an Olympus BX35 microscope. Image analysis detecting the total number of cells labelled by Hoechst and the number of Annexin V positive cells was performed using ImageJ (v. 1.52q).

### 2.6. Statistical Analysis

Statistical analysis was performed using the Student’s *t*-test for pairwise comparisons. A two-way repeated measures ANOVA followed by Tukey’s post hoc test was performed for multiple comparisons over time. A one-way ANOVA analysis followed by Tukey’s post hoc test was used for multiple comparisons at one time point. Significance is indicated in the figures as * *p* < 0.05, ** *p* < 0.01, *** *p* < 0.001, **** *p* < 0.0001. Statistical analyses were performed using GraphPad Prism 6 (GraphPad Holdings, San Diego, CA, USA). All samples were performed with at least *n* = 3 and expressed as an average with the standard error (SEM).

## 3. Results

### 3.1. NMed Optimisation

The first step in creating a targeted NMed system is to optimise the formulation to be stable and reproducible. Starting from an already established protocol for formulating PLGA NMeds, the physical characteristics (size, Z-potential, PDI, and weight yield%) were used to optimise the amount of the fluorophore-conjugated polymer (PLGA-Cy5 0.1–4% *w*/*w* to the total polymer amount) and % Pluronic^®^ F68 (0–3% *w*/*v*) used in the aqueous phase (Tables S1 and S2). The optimal values were chosen and held constant for all of the following experiments (0.2% PLGA-Cy5 and 1.5% Pluronic^®^ F68) due to the NMed characteristics (size ~157 nm, PDI 0.07, Z-potential −46 mV), reproducibility, and high weight yield percent (87%) (Table 1, first line). Regarding the residual amount of Pluronic^®^ F68 remaining in the matrix, no statistical differences were found when increasing the amount of surfactant used, remaining between 10–13% of the weight of the NMeds. This is in accordance with previously published results for PLGA-based NMeds and is an amount already demonstrated to be non-toxic (Table 1) [68,69].

**Figure 1:**
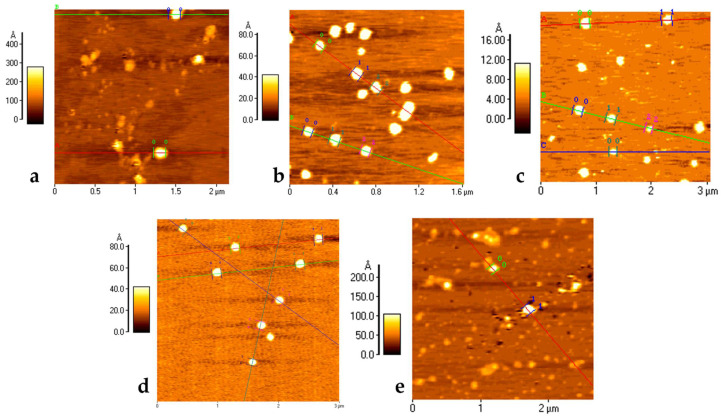
AFM Microscopy analysis of NMeds: (**a**) non-modified NMeds; (**b**) g7-NMeds; (**c**) PAAVF-NMeds; (**d**) M08J-NMeds; and (**e**) M08-NMeds. All images were obtained with NMeds modified with 10 µg of ligand. Coloured lines are added during post acquisition processing of images to analyse the size and height profile of NMeds, with numbered markers for each NMed.

With the matrix components established, the post-modification reaction to decorate the surface of the NMeds with each ligand was optimised. Mock reactions were performed to ensure that the peptide coupling reaction (EDC and NHS in MES 100 mM at pH 4.9) did not negatively influence the characteristics of the assembled NMeds. While the presence of the reagents and the buffer did not have any significant effect on the NMed characteristics, repetitive centrifugations led to a decreased weight yield from 90% to less than 30% with five cycles of centrifugation due to aggregation and poor resuspension (Table S3). To circumvent this problem, the first centrifugation step to purify the formed NMeds was removed, and the post-modification reaction was performed directly by adding concentrated 5× MES (500 mM) to the NMed formulation. This was possible because very little free PLGA was available, as suggested by the high weight yield (87%), and Pluronic^®^ F68 lacks any free amine or acid groups which could cause possible side reactions between the reagents and interfere in the surface modification. These reaction conditions had no significant effect on the size, PDI, or Z-potential of the NMeds (166 nm, 0.12, −32 mV) and, therefore, were used for surface modification with either 1 or 10 µg of each ligand (1 or 10 µg per 40 mg NMeds), which were then fully characterised. The addition of the different ligands had minor effects on the physical properties of the NMeds, maintaining the beneficial delivery characteristics ranging from 155 to 170 nm, PDI ~0.1, and a surface charge between −24 and −33 mV (Table 1). The high weight yields were maintained, although they exceeded 100%, which was probably due to residual salt from the reaction buffer that was not completely removed with the single centrifugation step. These physical characteristics were also supported by AFM microscopy, showing NMeds ranging from 100–200 nm, with good homogeneity and a spherical shape with no differences between the different ligand surface modifications (Figure 1).

Finally, with the intent to create stable drug-loaded NMeds, storage stability optimisation studies were performed. NMeds formed with these optimised parameters remained stable under numerous storage conditions over three weeks, including at 4 °C, under freeze-thaw conditions, and even when lyophilised (Table S4).

### 3.2. C6 Targeting Studies

**Figure 2:**
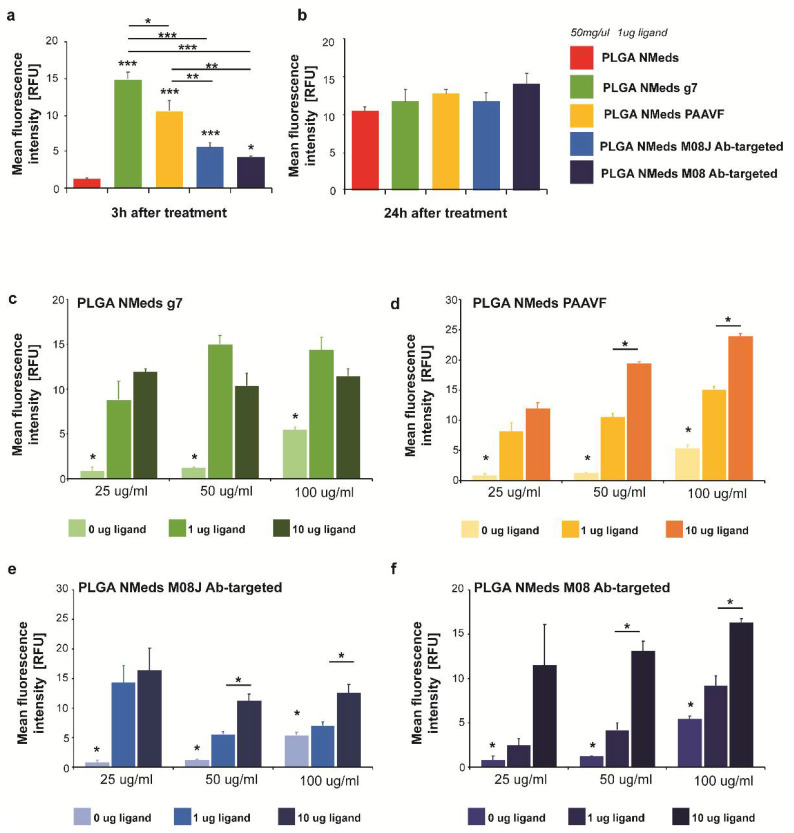
C6 cell uptake of 50 µg/mL targeted and non-targeted NMeds at (**a**) 3 h and (**b**) 24 h after administration. The relative fluorescence intensity of Cy5 intracellular signals (normalised to background fluorescence of untreated cells) was measured in 10 cells from *n* = 3 samples per condition. (**c**–**f**) Uptake of non-modified and targeted NMeds surfaced modified with 0, 1, or 10 µg of each ligand and administered at concentrations of 25, 50, or 100 µg/mL. The relative fluorescence intensity of Cy5 intracellular signals (normalised to background fluorescence of untreated cells) was measured in 10 cells from *n* = 3 samples per condition. Statistical analysis was performed with one-way ANOVA and post hoc test, * *p* < 0.5, ** *p* < 0.1, *** *p* < 0.01. Full statistical analysis is available in Supplementary material Table S5.

To investigate whether NMed uptake into GBM cells is ligand-dependent, targeting studies were performed using C6 GBM cell cultures. Imaging-based uptake studies were performed at both 3 and 24 h post addition of the fluorescent Cy5 containing NMeds (50 µg/mL) using the formulations with 1 µg of ligand in the post-modification reaction. Significant differences were observed even after only 3 h (Figure 2a), where all targeted NMeds, independent of the ligand, demonstrated higher cell uptake than the non-targeted control; however, after 24 h, the difference in targeted and non-targeted NMeds lost significance with all samples having similar uptake even though the total uptake increased compared to 3 h. This could be explained by the fact that NMeds dispersed in the cell medium were not removed and remained disposed to nonspecific uptake caused by the division and metabolism of the rapidly growing cells (Figure 2b).

To maximise targeted uptake and minimise the amount of non-specific cell internalisation, 3-h incubations were performed to test the dependence of ligand amount in the surface modification and NMed concentration on the uptake. NMeds modified with 0, 1, or 10 µg of each ligand were incubated for 3 h at concentrations of 25, 50, or 100 µg/mL (Figure 2c–f). As previously observed in the time course experiment, all targeted NMeds showed increased uptake over non-targeted controls independent of both ligand amount and NMed concentration. Three observable trends could be reported when comparing these two variables between the different ligand-modified NMeds (concentration and ligand). First, the uptake of g7-targeted NMeds neither increased depending on ligand amount in the reaction nor the total NMed concentration (Figure 2c). For the M08J-modified NMeds, increasing the concentration from 25 to 100 µg did not lead to statistically increased uptake; however, there was a statistical difference at higher NMed concentrations when the amount of ligand in the post-modification reaction was increased from 1 to 10 µg (Figure 2e). Lastly, PAAVF and M08-modified NMeds showed statistical increases in uptake dependent on both the ligand amount and NMed concentration (Figure 2d,f).

**Figure 3:**
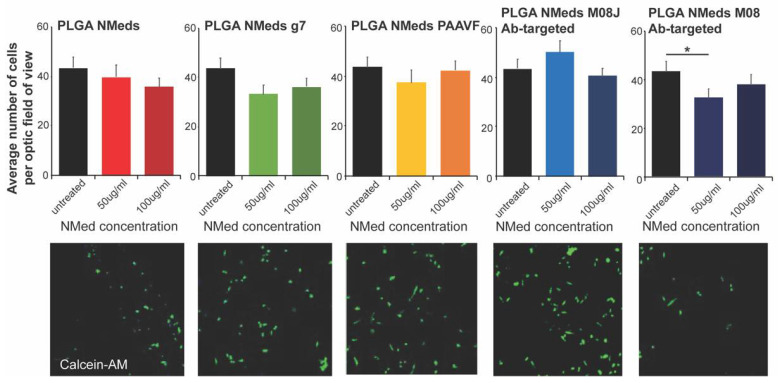
Cell growth inhibition studies. Quantification (**top**) of the number of cells per field analysed after incubating NMeds at a concentration of 50 or 100 µg/mL for 3 h and visualised by confocal microscopy (**bottom**). The number of cells visualised by DAPI and Calcein-AM staining per optic fields of view (OFV) was measured by counting *n* = 10 OFV for each condition. Statistical analysis was performed with one-way ANOVA and Post hoc analysis, * *p* < 0.05.

Further analysis of cell uptake determined the cell viability based on the number of cells remaining per field of vision (Figure 3). Cells were treated with 50 or 100 µg/mL of NMeds surface modified with 10 µg of each ligand. It was apparent that at these concentrations, the unmodified and targeted NMeds did not affect cell proliferation. The only exception was the M08 modified NMeds. At 100 µg/mL, no variation in cell number was seen. This was probably due to the presence of larger NMed aggregates that inhibited the uptake; however, at 50 µg/mL, M08 targeted NMeds led to a statistical decrease in cell number.

### 3.3. Co-Culture Studies

**Figure 4:**
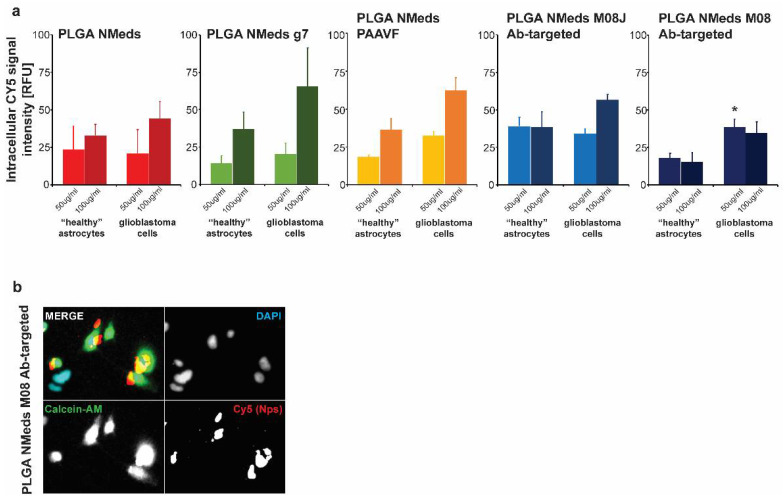
Cell uptake and growth inhibition studies. (**a**) Uptake in C6 (GBM) and DI TNC1 (“Healthy” astrocytes) cells after the administration of 50 and 100 µg/mL of targeted and non-targeted NMeds with 10 µg of each ligand after 3 h. Statistical analysis was performed with one-way ANOVA and Post hoc analysis, * *p* < 0.05; *n* = 8. (**b**) A representative image of M08 targeted NMeds, colocalizing with C6, but not DI TNC1 cells. Red: Cy5 (NMeds), blue: DAPI, green: Calcein-AM.

To investigate whether the ligands facilitated selective uptake into GBM cells, co-culture experiments were performed using both rat C6 GBM cells that are derived from astrocytes and “healthy” rat DI TNC1 astrocytes. The C6 GBM cells were labelled with Calcein-AM in the co-culture to distinguish the difference between the cells. The fluorescence of internalised NMeds in the co-cultured cells demonstrated that non-targeted, g7 and M08J-targeted NMeds showed comparable levels at 50 or 100 µg/mL. The similar results between non-targeted and targeted NMeds indicate a natural and non-specific uptake of these NMeds into both cell types that is not dependent on targeting. There was a slight, but not statistically significant, increase in C6 cell uptake of AAVF-targeted NMeds. Uniquely M08 targeted NMeds showed a statistically increased uptake into GBM cells over healthy astrocytes (Figure 4a,b).

**Figure 5:**
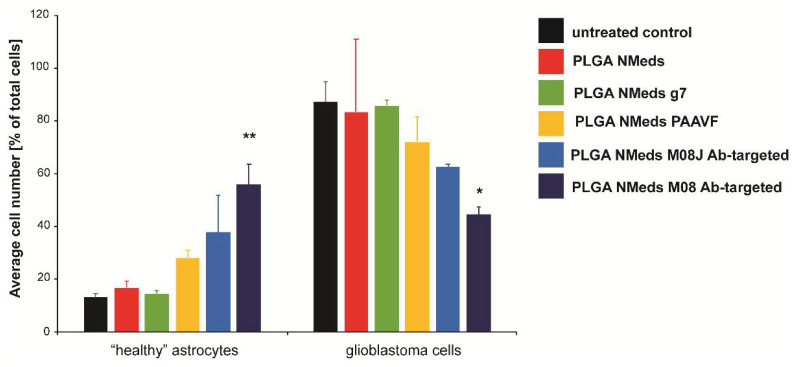
The confocal microscopy quantification ofC6 and DI TNC1 cell numbers per optic field of view (OFV) after a 3 h incubation with 50 µg/mL of NMeds modified with 10 µg of ligand. All experiments were performed in triplicate (*n* = 3), and the number of cells visualised by DAPI and Calcein-AM staining per OFV was measured by counting 8 OFV for each condition. Statistical analysis was performed with one-way ANOVA, * *p* < 0.05, ** *p* < 0.01.

Moreover, the experiment was repeated to analyse the total number of each cell type (Figure 5). Untreated co-culture wells showed that the cell growth between the two cell lines led to a ~80:20 ratio of C6:DI TNC1 cells. This could be explained by the faster cell division rate of C6 cells over healthy astrocytes and the potentially invasive nature of C6 cells, which could lead to them spreading out and occupying more surface space in the well, “choking out” astrocyte growth. This growth ratio was maintained with non-targeted, AAVF, and g7-targeted NMeds, suggesting no effect on cell proliferation; however, M08J targeted NMeds did show a difference. Here, the “healthy” astrocytes increased to up to 40%, with a corresponding decrease in C6 cells to 60%.

Nevertheless, the most potent effects were seen with M08 targeted NMeds, which inverted this ratio with “healthy” astrocytes constituting 60% of the culture with only 40% GBM cells. Supporting other literature for CSV antibodies [54], this result demonstrates a biological effect of these antibodies to decrease GBM growth and allowing the healthy astrocytes to grow more freely. This effect could further promote the delivery and efficacy of anticancer therapeutics using these types of targeted NMeds.

### 3.4. Chemotherapeutic Drug Delivery with Targeted NMeds

**Figure 6:**
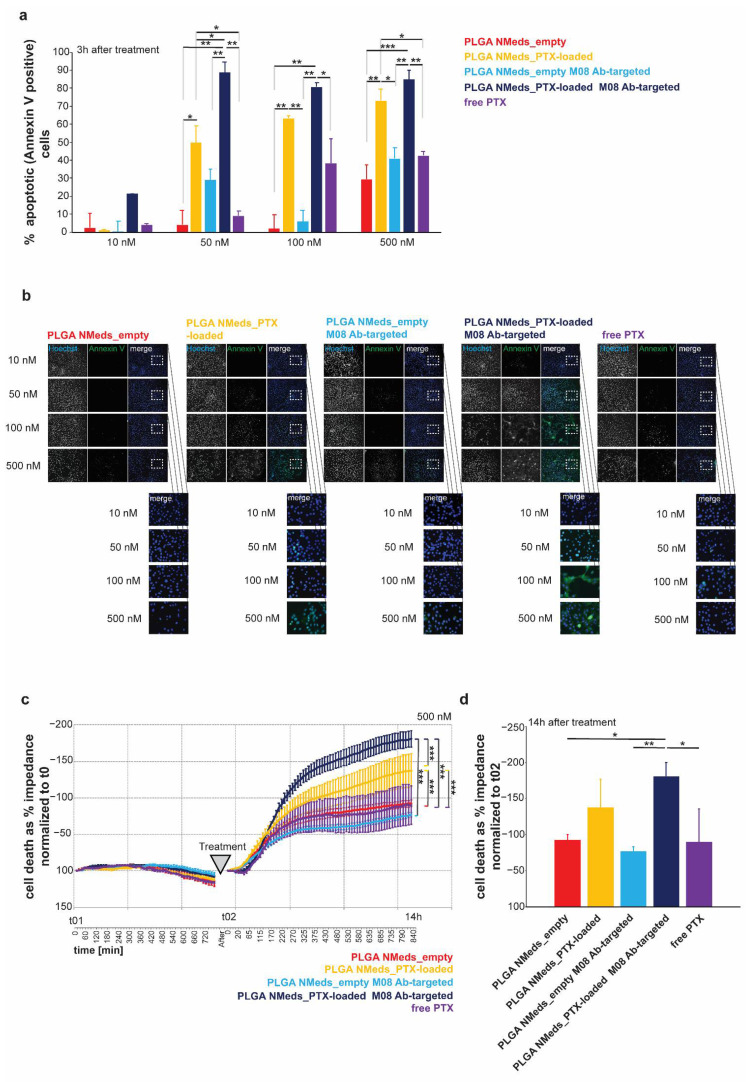
Effect of PTX-loaded NMeds on cell health of C6 glioblastoma cells. (**a**) Quantification of apoptosis using FITC-Annexin V. The percent of Annexin V positive cells of total cells is shown as average from *n* = 3 OFV. C6 cells were treated for 3 h with empty non-targeted and M08-targeted NMeds, and non-targeted and M08-targeted NMeds delivering the equivalent of 10, 50, 100, and 500 nM PTX. Free PTX at the same concentrations was used as controls. (**b**) Representative images showing all cells. The visualisation was achieved by labelling the nuclei with Hoechst staining and labelling apoptotic cells with Annexin V-FITC. (**c**,**d**) Cell impedance measurement after treatment with empty non-targeted and M08-targeted NMeds, as well as their PTX loaded counterparts (the equivalent of 500 nM PTX) and compared with500 nM free PTX as a control treatment. Before treatment, cells were grown for 12.5 h (t0_1_—750 min). *n* = 3 per treatment. Cell death was evaluated as a decrease in impedance and shown as % of t0. (**d**) Cell death analysis at the 14 h endpoint of the treatment shown in (**c**). One-way ANOVA analysis and Tukey’s Post hoc tests were performed, * *p* < 0.05, ** *p* < 0.01, *** *p* < 0.001.

Finally, if the GBM-specific delivery of targeted NMeds loaded with the chemotherapeutic drug paclitaxel (PTX) were tested compared to free drug. In the first set of experiments, C6 GBM cells were treated with NMeds delivering the equivalent of 10, 50, 100, and 500 nM with the same concentrations of free PTX used as controls-. By analysing FITC-Annexin V as an apoptotic cell marker, PTX-loaded NMeds (targeted and non-targeted) induced higher apoptosis rates than free PTX. Furthermore, M08-targeted NMeds outperformed free PTX and led to an increased number of apoptotic cells compared to non-targeted NMeds. While the toxicity of free PTX significantly increased from 50 to 500 nM, no further increase was observed for PTX-loaded M08-NMeds even at low concentrations, which already reached almost 100% of apoptotic cells at 50 nM. These results indicate the high therapeutic potential of these targeted NMeds, which are much more toxic (100% compared to 40%) even at drug concentrations 10× lower than the free drug (Figure 6a,b).

Next, a label-free cell health assay based on impedance measurements was performed to understand whether the increase in apoptotic cells translates to an increase in cell death (Figure 6c,d). C6 GBM cells were treated with NMeds delivering the equivalent of 500 nM PTX and compared to 500 nM of free PTX since a significant effect of free PTX was previously seen at this concentration. Cells were seeded and grown until confluent and monitored for 12.5 h before treatment (Figure 6c). Cell impedance was measured every 15 min, and cell death was evaluated as a decrease in impedance. Before treatment, no significant difference between cells assigned to different treatment groups was observed. Cells were then treated with NMeds or free PTX and monitored for another 14 h. A significant effect of treatment, time, and treatment x time interaction was observed comparing empty with PTX-loaded NMeds. Non-targeted and M08 PTX-loaded NMeds induced significantly more cell death than empty NMeds; however, over the time course, M08 targeted PTX-loaded M08 NMeds were significantly more toxic than all other samples, including the non-targeted PTX-loaded NMeds. This difference remained significant even at the endpoint measurement (Figure 6d).

## 4. Discussion

GBM is one of the most aggressive forms of brain cancer that affects thousands of people and is notable for its poor prognoses. Since current strategies lack specificity, which leads to toxicity in healthy tissues and does not inhibit recurrences, targeted nanomedicines (NMeds) are being widely investigated as promising new tools, as demonstrated by an abundance of reviews in the last two years [70,71,72,73,74,75,76]. One of the most important aspects to consider when optimising a targeted NMed is selecting an appropriate targeting ligand. In general, targeting strategies fall under three main categories [77,78]. Cell-penetrating peptides represent one first approach [79,80,81,82]. These peptides help deliver pharmaceutics into cancer cells but are often considered non-specific, as they promote indiscriminate NMed uptake. Secondly, upregulated cell pathways can be targeted [83,84,85], for example, using miRNAs that disrupt those pathways [86,87]. Similarly, the tumour microenvironment can be a site of therapeutic interest [88,89,90,91,92]: one example is exploiting the high concentration of Reactive Oxygen Species (ROS) with ROS-sensitive NMeds that would release the drug only in the presence of ROS [93,94]. However, major limitations to these strategies lay in the poor stability of sensitive molecules, such as RNAs and the lack of cell specificity that could still lead to off-target effects.

The third strategy, and the focus of this work, consists in using cell-specific ligands, which take advantage of upregulated receptors on the cell surface. Due to the variations in the biochemical identity of tumour cells, a myriad of reviews and research papers have been dedicated to the different ligands for GBM targeting, ranging from small molecules to peptides to antibodies [77,95,96,97,98,99]. While having so many options is beneficial, and this targeting strategy is generally more effective compared to the previous two, it is important to remember that many of these have characteristics that could still limit their use, such as poor specificity, low affinity, incapacity to internalise or activate the receptor with nano-sized cargo, saturability etc. [100,101]. Thus, it is crucial to investigate novel ligands that can improve the GBM-specific delivery of therapeutics. In this study, polymeric NMeds were surface coated with different ligands, peptides and antibodies and evaluated for their GBM targeting potential.

First, the standardisation of the formulation process to give reproducible and consistent NMeds was critical to be able to focus on the targeting ligand effects and avoid any differences due to size, morphology, or surface charge. NMeds modified with each of the four ligands showed a rapid and improved uptake in GBM cells even at short periods. Different trends in the NMed uptake arose when analysing the impact of the amount of ligand on the surface, or the concentration of NMeds, evidencing a unique behaviour for each ligand. Overall, the only ligand that showed GBM specificity over DI TNC1 cells was the antibody M08. M08 not only improved GBM specific uptake, but also showed potential biological activity: analysing cell growth, M08-targeted NMeds led to decreased GBM cell growth in a single culture, while in co-culture, they did not affect the astrocytes, allowing them to grow more readily. When loaded with the chemotherapeutic PTX, higher cell death was observed after dosing M08-NMeds over both free PTX and non-targeted NMeds in GBM cells; also, at only 3 h of incubation, M08-targeted NMeds significantly increased apoptotic markers in the cells, highlighting the anti-cancer potential of these systems. These results show the promise of a well optimised targeted NMed. At the same time, CSV is known to be upregulated to different extent in different cell lines, with C6 cells having a lower amount compared to other models [102,103]. Hence, these results could also evidence a severe underestimation of the true potential of CSV targeted NMeds.

Our results can be compared to a recent study by Ullah et al. [104] where PTX-loaded NMeds were surface modified with the cell-penetrating peptide RGD. This ligand is known to have specificity towards brain cancer, where the cells upregulate integrin αvβ3 [82], and has been widely used to help deliver and increase the potency of chemotherapeutics, such as temozolomide or PTX [105,106,107]. In their study, RGD-NMeds showed a similar effect to our M08 modified NMeds in the arrest of cell growth, but the effect was solely due to the presence of PTX: in fact, there was no significant difference in cell viability between free drug, non-targeted NMeds, and RGD-targeted NMeds. Furthermore, when analysing cell toxicity, no significant difference was evidenced between the free drug and RGD-targeted NMeds, even if the latter showed decreased tumour volumes when dosed in vivo. In the present study, the presence of M08 on the NMed surface led to higher PTX toxicity compared both to free drug and non-targeted NMeds, indicating improved uptake and enhanced synergistic effects of the drug and M08 when co-delivered in vitro.

Another point to address when considering GBM-targeted NMeds is distinguishing between targeting ligands that promote BBB crossing and others that improve GBM uptake [108,109,110]. Since the BBB is a major limitation to the effectiveness of brain cancer treatments, BBB-targeted NMeds are often effective in reducing tumour volume, but they lack specificity for diseased cells. For this reason, although not specific for GBM, NMeds decorated with g7 and AAVF can still be valuable players in the search for improved GBM treatments, as they are known to be effective for BBB crossing [43,45,111,112]. This brings up a major point in the field, described in great detail in a review by Luo et al. [113]. Here, the authors highlight that BBB-targeted NMeds are often considered GBM specific, but single targeting strategies cannot guarantee both the BBB infiltration and GBM specificity. Thus, various NMeds with two or more targeting ligands on their surface or bispecific antibodies are currently being investigated [114,115,116]. Although these systems show improved efficacy, combining several targeting ligands on the same NMed drastically increase the difficulty of production and characterisation [117,118]. Considering these issues, the M08 antibody can represent a step forward in the design of GBM-targeted NMeds. From our results, it is evident that M08 is a prime candidate thanks to its ability to cross the BBB, specifically target GBM cells, and exert biological anti-cancer activity. Furthermore, all these functions in a single antibody make producing and characterising these systems easier, leading to better chances of translating these results into preclinical applications.

## 5. Conclusions

NMeds are one of the most promising medical tools for difficult-to-treat diseases, such as GBM. Enhancing the solubility, biodistribution, and compatibility with chemotherapeutics are significant players in designing treatments; however, to have an effective NMed treatment against GBM, selective delivery into the brain and only to GBM cells is critical to lower doses, improve drug delivery, and lower collateral damage to healthy surrounding cells. This study demonstrated several promising ligands with the potential for BBB and/or GBM targeting. In addition, by optimising surface modifications, we have indicated a monoclonal antibody (M08) that can specifically enter GBM cells over healthy cells and cause synergistic effects when delivering the chemotherapeutic PTX. This improved cell uptake and GBM specificity is an important step to creating improved chemotherapeutic NMeds that could offer higher curative potential with lowered off-target toxicity to healthy cells.

## Figures and Tables

**Table 1:** Physico-chemical characteristics of ligand modified NMeds. Results are given as the median plus/minus the standard deviation (SD).

NMed Formulation	Ligand Amount (µg)	Size (nm)	PDI	Z Potential (mV)	% Residual Surfactant	% Weight Yield
Optimised NMed	0	157 ± 8	0.07 ± 0.01	−45.6 ± 4	12 ± 5	87 ± 9
NMeds with mock reaction	0	166 ± 10	0.12 ± 0.01	−33 ± 10	10 ± 7	93 ± 6
g7-NMeds	1	155 ± 13	0.09 ± 0.03	−34 ± 11	9 ± 8	92 ± 5
PAAVF-NMeds		156 ± 9	0.08 ± 0.01	−25 ± 9	10 ± 5	112 ± 10
M08J-NMeds		161 ± 11	0.11 ± 0.02	−31 ± 8	11 ± 5	102 ± 7
M08-NMeds		164 ± 10	0.16 ± 0.01	−26 ± 10	11 ± 6	97 ± 9
g7-NMeds	10	159 ± 10	0.08 ± 0.01	−31 ± 13	8 ± 9	104 ± 5
PAAVF-NMeds		156 ± 12	0.09 ± 0.02	−29 ± 8	10 ± 5	96 ± 3
M08J-NMeds		159 ± 11	0.10 ± 0.02	−29 ± 11	9 ± 9	110 ± 4
M08-NMeds		160 ± 14	0.18 ± 0.03	−32 ± 9	11 ± 6	119 ± 4

## Data Availability

All data may be requested directly from the corresponding author.

## Supplementary Materials

The following supporting information can be downloaded at: https://www.mdpi.com/article/10.3390/pharmaceutics14071450/s1, Table S1: Physico-chemical characteristics of NMeds formulated with different percentages of PLGA-CY5; Table S2: The effects of Pluronic^®^ F68 amount on physico-chemical characteristics of NMeds; Table S3: Physico-chemical characteristics of NMeds after mock reaction, submitted to several cycles of centrifugation; Table S4: Stability results of non-modified NMeds and targeted NMeds after 3 weeks after storage at 4–8 °C, freezing at −20 °C without trehalose, freezing at −20 °C with trehalose at 3 *w*/*w* ratio and after lyophilisation. Table S5: Full statistical data for the in vitro results.
