# Supplementary material for: Glioblastoma Multiforme Selective Nanomedicines for Improved Anti-Cancer Treatments
Source: Pharmaceutics. 2022 Jul 12;14(7):1450. doi: 10.3390/pharmaceutics14071450 (PMC9325049; doi:10.3390/pharmaceutics14071450)
Supplement: Supplementary file 1 [file pharmaceutics-14-01450-s001.zip › pharmaceutics-1778218-supplementary.pdf]

Table S1. Physico-chemical characteristics of NMeds formulated with different percentages of PLGA-Cy5.

PLGA-CY5 (% w/w)	Size (nm)	PDI	Z-potential (mV)	% Weight yield
4	142 ± 23	0.21 ± 0.01	-28.8 ± 12	28 ± 15
2	157 ± 15	0.07 ± 0.01	-35.6± 10	34 ± 11
1	160 ± 25	0.13 ± 0.01	-28.4 ± 4	41 ± 13
0.4	156 ± 20	0.08 ± 0.02	-30.2 ± 7	82 ± 6
0.2	147 ± 18	0.06 ± 0.01	-31.1 ± 3	90 ± 8
0.1	155 ± 12	0.19 ± 0.01	-31.5 ± 5	93 ± 8

Table S2. The effects of Pluronic® F68 amount on physico-chemical characteristics of NMeds.

NMed formulation	% w/v surfactant in Aqueous phase (12.5 mL)	Size (nm)	PDI	Z-potential (mV)	% Residual surfactant	% Weight yield
1	MilliQ water	183 ± 12	0.14 ± 0.01	-43.8 ± 5	n/a	25 ± 8
2	0.5%	155 ± 7	0.08 ± 0.02	-39.4 ± 6	10 ± 3	42 ± 10
3	1.5%	157 ± 8	0.07 ± 0.01	-45.6 ± 4	12 ± 5	87 ± 9
4	3%	183 ± 10	0.20 ± 0.01	-45.7 ± 7	14 ± 7	88 ± 4

Table S3. Physico-chemical characteristics of NMeds after mock reaction, submitted to several cycles of centrifugation.

Centrifugation	Size (nm)	PDI	Z potential (mV)	% Weight yield
1*	147 ± 18	0.05 ± 0.01	-31 ± 3	90 ± 8
2	166 ± 27	0.12 ± 0.01	-32 ± 7	60 ± 7
3	170 ± 15	0.05 ± 0.01	-31 ± 8	43 ± 12
4	160 ± 10	0.18 ± 0.03	-29 ± 9	30 ± 15
5	156 ± 11	0.08 ± 0.01	-25 ± 5	27 ± 13
1**	166 ± 10	0.12 ± 0.01	-32 ± 5	90 ± 13

* Centrifugation step after NMed formulation, followed by mock reaction without any further purification. Sample analysed in reaction buffer.

** Post-modification was run on NMeds directly from the formulation without purification. One centrifugation step was performed on the NMeds after the mock reaction.

Table S4. Stability results of non-modified NMeds and targeted NMeds after 3 weeks after storage at 4 - 8°C, freezing at -20°C without trehalose, freezing at -20 °C with trehalose at 3 w/w ratio and after lyophilisation.

NMed formulation	Storage conditions	Size (nm)	PDI	Z potential (mV)
non-modified NMeds	Storage 4 - 8°C	289 ± 10	0.29 ± 0.01	-28 ± 11
g7-NMeds		260 ± 14	0.33 ± 0.03	-22.6 ± 8
PAAVF-NMeds		180 ± 9	0.25 ± 0.03	-27.4 ± 6
M08J-NMeds		176 ± 11	0.19 ± 0.01	-27.6 ± 9
M08-NMeds		220 ± 10	0.26 ± 0.02	-18.1 ± 8
Non-modified NMeds	Freezing -20°C without trehalose	560 ± 60	0.66 ± 0.04	-10.8 ± 8
g7-NMeds		340 ± 54	0.33 ± 0.04	-28.7 ± 9
PAAVF-NMeds		176 ± 9	0.13 ± 0.02	-29.1 ± 6
M08J-NMeds		182 ± 8	0.17 ± 0.03	-27.1 ± 11
M08-NMeds		230 ± 10	0.28 ± 0.02	-28.7 ± 10

Non-modified NMeds	Freezing -20°C with trehalose at 3 w/w ratio	254 ± 10	0.33 ± 0.02	-25.0 ± 6
g7-NMeds		228 ± 8	0.21 ± 0.01	-25.6 ± 8
PAAVF-NMeds		181 ± 9	0.12 ± 0.01	-31.9 ± 5
M08J-NMeds		179 ± 11	0.18 ± 0.02	-30.4 ± 6
M08-NMeds		211 ± 14	0.19 ± 0.02	-30.8 ± 6
Non-modified NMeds	Lyophilisation	350 ± 8	0.42 ± 0.03	-20.4 ± 8
g7-NMeds		288 ± 11	0.30 ± 0.02	-18.8 ± 13
PAAVF-NMeds		189 ± 12	0.23 ± 0.01	-17.2 ± 13
M08J-NMeds		202 ± 9	0.24 ± 0.02	-22.4 ± 12
M08-NMeds		240 ± 14	0.25 ± 0.01	-21.4 ± 12

Table S5. Full statistical data for the *in vitro* results.

2-way ANOVA: effect of concentration, effect of treatment, concentration x treatment interaction		
Source of Variation	% of total variation	P value
Interaction	14.97	<0.0001
concentration	32.58	<0.0001
treatment	49.28	<0.0001

**10 mM**	
ANOVA summary	
F	5.157
P value	0.0507
P value summary	ns
Significant diff. among means (P < 0.05)?	No

50 mM			
ANOVA summary			
F	26.27		
P value	0.0015		
P value summary	**		
Significant diff. among means (P < 0.05)?	Yes		
R squared	0.9546		
Tukey's multiple comparisons test	Significant?	Summary	Adjusted P Value
PLGA NPS_EMPTY vs. PLGA NPS_PTX-LOADED	Yes	*	0.0248
PLGA NPS_EMPTY vs. PLGA NPS_EMPTY M08 AB-TARGETED	No	ns	0.2018
PLGA NPS_EMPTY vs. PLGA NPS_PTX-LOADED M08 AB-TARGETED	Yes	**	0.0016
PLGA NPS_EMPTY vs. PTX	No	ns	0.9819
PLGA NPS_PTX-LOADED vs. PLGA NPS_EMPTY M08 AB-TARGETED	No	ns	0.3155
PLGA NPS_PTX-LOADED vs. PLGA NPS_PTX-LOADED M08 AB-TARGETED	Yes	*	0.0467
PLGA NPS_PTX-LOADED vs. PTX	Yes	*	0.0389
PLGA NPS_EMPTY M08 AB-TARGETED vs. PLGA NPS_PTX-LOADED M08 AB-TARGETED	Yes	**	0.008
PLGA NPS_EMPTY M08 AB-TARGETED vs. PTX	No	ns	0.3437
PLGA NPS_PTX-LOADED M08 AB-TARGETED vs. PTX	Yes	**	0.0021

100 mM			
ANOVA summary			
F	27.04		
P value	0.0014		
P value summary	**		
Significant diff. among means (P < 0.05)?	Yes		
R squared	0.9558		
Tukey's multiple comparisons test	Significant?	Summary	Adjusted P Value
PLGA NPS_EMPTY vs. PLGA NPS_PTX-LOADED	Yes	**	0.0067
PLGA NPS_EMPTY vs. PLGA NPS_EMPTY M08 AB-TARGETED	No	ns	0.9921
PLGA NPS_EMPTY vs. PLGA NPS_PTX-LOADED M08 AB-TARGETED	Yes	**	0.0022
PLGA NPS_EMPTY vs. PTX	No	ns	0.0579
PLGA NPS_PTX-LOADED vs. PLGA NPS_EMPTY M08 AB-TARGETED	Yes	**	0.009
PLGA NPS_PTX-LOADED vs. PLGA NPS_PTX-LOADED M08 AB-TARGETED	No	ns	0.4452
PLGA NPS_PTX-LOADED vs. PTX	No	ns	0.1921
PLGA NPS_EMPTY M08 AB-TARGETED vs. PLGA NPS_PTX-LOADED M08 AB-TARGETED	Yes	**	0.0027
PLGA NPS_EMPTY M08 AB-TARGETED vs. PTX	No	ns	0.0863
PLGA NPS_PTX-LOADED M08 AB-TARGETED vs. PTX	Yes	*	0.0321

500 mM				
ANOVA summary				

F	37.35			
P value	0.0006			
P value summary	***			
Significant diff. among means (P < 0.05)?	Yes			
R squared	0.9676			

Tukey's multiple comparisons test	Significant?	Summary	Adjusted P Value
PLGA NPS_EMPTY vs. PLGA NPS_PTX-LOADED	Yes	**	0.0026
PLGA NPS_EMPTY vs. PLGA NPS_EMPTY M08 AB-TARGETED	No	ns	0.3416
PLGA NPS_EMPTY vs. PLGA NPS_PTX-LOADED M08 AB-TARGETED	Yes	***	0.0009
PLGA NPS_EMPTY vs. PTX	No	ns	0.2479
PLGA NPS_PTX-LOADED vs. PLGA NPS_EMPTY M08 AB-TARGETED	Yes	*	0.0103
PLGA NPS_PTX-LOADED vs. PLGA NPS_PTX-LOADED M08 AB-TARGETED	No	ns	0.3312
PLGA NPS_PTX-LOADED vs. PTX	Yes	*	0.013
PLGA NPS_EMPTY M08 AB-TARGETED vs. PLGA NPS_PTX-LOADED M08 AB-TARGETED	Yes	**	0.0026
PLGA NPS_EMPTY M08 AB-TARGETED vs. PTX	No	ns	0.9972
PLGA NPS_PTX-LOADED M08 AB-TARGETED vs. PTX	Yes	**	0.0031

**Tukey's multiple comparisons test**	**Significant?**	**Summary**	**Adjusted P Value**
10:PLGA NPS_EMPTY vs. 10:PLGA NPS_PTX-LOADED	No	ns	>0.9999
10:PLGA NPS_EMPTY vs. 10:PLGA NPS_EMPTY M08 AB-TARGETED	No	ns	>0.9999
10:PLGA NPS_EMPTY vs. 10:PLGA NPS_PTX-LOADED M08 AB-TARGETED	No	ns	0.6288
10:PLGA NPS_EMPTY vs. 10:PTX	No	ns	>0.9999
10:PLGA NPS_EMPTY vs. 50:PLGA NPS_EMPTY	No	ns	>0.9999
10:PLGA NPS_EMPTY vs. 50:PLGA NPS_PTX-LOADED	Yes	***	0.0006
10:PLGA NPS_EMPTY vs. 50:PLGA NPS_EMPTY M08 AB-TARGETED	No	ns	0.1524
10:PLGA NPS_EMPTY vs. 50:PLGA NPS_PTX-LOADED M08 AB-TARGETED	Yes	****	<0.0001
10:PLGA NPS_EMPTY vs. 50:PTX	No	ns	>0.9999
10:PLGA NPS_EMPTY vs. 100:PLGA NPS_EMPTY	No	ns	>0.9999
10:PLGA NPS_EMPTY vs. 100:PLGA NPS_PTX-LOADED	Yes	****	<0.0001
10:PLGA NPS_EMPTY vs. 100:PLGA NPS_EMPTY M08 AB-TARGETED	No	ns	>0.9999
10:PLGA NPS_EMPTY vs. 100:PLGA NPS_PTX-LOADED M08 AB-TARGETED	Yes	****	<0.0001
10:PLGA NPS_EMPTY vs. 100:PTX	Yes	*	0.0146
10:PLGA NPS_EMPTY vs. 500:PLGA NPS_EMPTY	No	ns	0.1379
10:PLGA NPS_EMPTY vs. 500:PLGA NPS_PTX-LOADED	Yes	****	<0.0001
10:PLGA NPS_EMPTY vs. 500:PLGA NPS_EMPTY M08 AB-TARGETED	Yes	**	0.0071
10:PLGA NPS_EMPTY vs. 500:PLGA NPS_PTX-LOADED M08 AB-TARGETED	Yes	****	<0.0001
10:PLGA NPS_EMPTY vs. 500:PTX	Yes	**	0.0045

10:PLGA NPS_PTX-LOADED vs. 10:PLGA NPS_EMPTY M08 AB-TARGETED	No	ns	>0.9999
10:PLGA NPS_PTX-LOADED vs. 10:PLGA NPS_PTX-LOADED M08 AB-TARGETED	No	ns	0.5266
10:PLGA NPS_PTX-LOADED vs. 10:PTX	No	ns	>0.9999
10:PLGA NPS_PTX-LOADED vs. 50:PLGA NPS_EMPTY	No	ns	>0.9999
10:PLGA NPS_PTX-LOADED vs. 50:PLGA NPS_PTX-LOADED	Yes	***	0.0004
10:PLGA NPS_PTX-LOADED vs. 50:PLGA NPS_EMPTY M08 AB-TARGETED	No	ns	0.1127
10:PLGA NPS_PTX-LOADED vs. 50:PLGA NPS_PTX-LOADED M08 AB-TARGETED	Yes	****	<0.0001
10:PLGA NPS_PTX-LOADED vs. 50:PTX	No	ns	0.9998
10:PLGA NPS_PTX-LOADED vs. 100:PLGA NPS_EMPTY	No	ns	>0.9999
10:PLGA NPS_PTX-LOADED vs. 100:PLGA NPS_PTX-LOADED	Yes	****	<0.0001
10:PLGA NPS_PTX-LOADED vs. 100:PLGA NPS_EMPTY M08 AB-TARGETED	No	ns	>0.9999
10:PLGA NPS_PTX-LOADED vs. 100:PLGA NPS_PTX-LOADED M08 AB-TARGETED	Yes	****	<0.0001
10:PLGA NPS_PTX-LOADED vs. 100:PTX	Yes	*	0.0103
10:PLGA NPS_PTX-LOADED vs. 500:PLGA NPS_EMPTY	No	ns	0.1016
10:PLGA NPS_PTX-LOADED vs. 500:PLGA NPS_PTX-LOADED	Yes	****	<0.0001
10:PLGA NPS_PTX-LOADED vs. 500:PLGA NPS_EMPTY M08 AB-TARGETED	Yes	**	0.005
10:PLGA NPS_PTX-LOADED vs. 500:PLGA NPS_PTX-LOADED M08 AB-TARGETED	Yes	****	<0.0001
10:PLGA NPS_PTX-LOADED vs. 500:PTX	Yes	**	0.0031
10:PLGA NPS_EMPTY M08 AB-TARGETED vs. 10:PLGA NPS_PTX-LOADED M08 AB-TARGETED	No	ns	0.4544
10:PLGA NPS_EMPTY M08 AB-TARGETED vs. 10:PTX	No	ns	>0.9999
10:PLGA NPS_EMPTY M08 AB-TARGETED vs. 50:PLGA NPS_EMPTY	No	ns	>0.9999
10:PLGA NPS_EMPTY M08 AB-TARGETED vs. 50:PLGA NPS_PTX-LOADED	Yes	***	0.0003
10:PLGA NPS_EMPTY M08 AB-TARGETED vs. 50:PLGA NPS_EMPTY M08 AB-TARGETED	No	ns	0.0897
10:PLGA NPS_EMPTY M08 AB-TARGETED vs. 50:PLGA NPS_PTX-LOADED M08 AB-TARGETED	Yes	****	<0.0001
10:PLGA NPS_EMPTY M08 AB-TARGETED vs. 50:PTX	No	ns	0.9993
10:PLGA NPS_EMPTY M08 AB-TARGETED vs. 100:PLGA NPS_EMPTY	No	ns	>0.9999
10:PLGA NPS_EMPTY M08 AB-TARGETED vs. 100:PLGA NPS_PTX-LOADED	Yes	****	<0.0001
10:PLGA NPS_EMPTY M08 AB-TARGETED vs. 100:PLGA NPS_EMPTY M08 AB-TARGETED	No	ns	>0.9999
10:PLGA NPS_EMPTY M08 AB-TARGETED vs. 100:PLGA NPS_PTX-LOADED M08 AB-TARGETED	Yes	****	<0.0001
10:PLGA NPS_EMPTY M08 AB-TARGETED vs. 100:PTX	Yes	**	0.0079
10:PLGA NPS_EMPTY M08 AB-TARGETED vs. 500:PLGA NPS_EMPTY	No	ns	0.0806
10:PLGA NPS_EMPTY M08 AB-TARGETED vs. 500:PLGA NPS_PTX-LOADED	Yes	****	<0.0001
10:PLGA NPS_EMPTY M08 AB-TARGETED vs. 500:PLGA NPS_EMPTY M08 AB-TARGETED	Yes	**	0.0038
10:PLGA NPS_EMPTY M08 AB-TARGETED vs. 500:PLGA NPS_PTX-LOADED M08 AB-TARGETED	Yes	****	<0.0001
10:PLGA NPS_EMPTY M08 AB-TARGETED vs. 500:PTX	Yes	**	0.0024
10:PLGA NPS_PTX-LOADED M08 AB-TARGETED vs. 10:PTX	No	ns	0.7427

10:PLGA NPS_PTX-LOADED M08 AB-TARGETED vs. 50:PLGA NPS_EMPTY	No	ns	0.7618
10:PLGA NPS_PTX-LOADED M08 AB-TARGETED vs. 50:PLGA NPS_PTX-LOADED	No	ns	0.0977
10:PLGA NPS_PTX-LOADED M08 AB-TARGETED vs. 50:PLGA NPS_EMPTY M08 AB-TARGETED	No	ns	0.9999
10:PLGA NPS_PTX-LOADED M08 AB-TARGETED vs. 50:PLGA NPS_PTX-LOADED M08 AB-TARGETED	Yes	****	<0.0001
10:PLGA NPS_PTX-LOADED M08 AB-TARGETED vs. 50:PTX	No	ns	0.9784
10:PLGA NPS_PTX-LOADED M08 AB-TARGETED vs. 100:PLGA NPS_EMPTY	No	ns	0.5958
10:PLGA NPS_PTX-LOADED M08 AB-TARGETED vs. 100:PLGA NPS_PTX-LOADED	Yes	**	0.0027
10:PLGA NPS_PTX-LOADED M08 AB-TARGETED vs. 100:PLGA NPS_EMPTY M08 AB-TARGETED	No	ns	0.8691
10:PLGA NPS_PTX-LOADED M08 AB-TARGETED vs. 100:PLGA NPS_PTX-LOADED M08 AB-TARGETED	Yes	****	<0.0001
10:PLGA NPS_PTX-LOADED M08 AB-TARGETED vs. 100:PTX	No	ns	0.7729
10:PLGA NPS_PTX-LOADED M08 AB-TARGETED vs. 500:PLGA NPS_EMPTY	No	ns	0.9998
10:PLGA NPS_PTX-LOADED M08 AB-TARGETED vs. 500:PLGA NPS_PTX-LOADED	Yes	***	0.0002
10:PLGA NPS_PTX-LOADED M08 AB-TARGETED vs. 500:PLGA NPS_EMPTY M08 AB-TARGETED	No	ns	0.5701
10:PLGA NPS_PTX-LOADED M08 AB-TARGETED vs. 500:PLGA NPS_PTX-LOADED M08 AB-TARGETED	Yes	****	<0.0001
10:PLGA NPS_PTX-LOADED M08 AB-TARGETED vs. 500:PTX	No	ns	0.4399
10:PTX vs. 50:PLGA NPS_EMPTY	No	ns	>0.9999
10:PTX vs. 50:PLGA NPS_PTX-LOADED	Yes	***	0.0009
10:PTX vs. 50:PLGA NPS_EMPTY M08 AB-TARGETED	No	ns	0.2116
10:PTX vs. 50:PLGA NPS_PTX-LOADED M08 AB-TARGETED	Yes	****	<0.0001
10:PTX vs. 50:PTX	No	ns	>0.9999
10:PTX vs. 100:PLGA NPS_EMPTY	No	ns	>0.9999
10:PTX vs. 100:PLGA NPS_PTX-LOADED	Yes	****	<0.0001
10:PTX vs. 100:PLGA NPS_EMPTY M08 AB-TARGETED	No	ns	>0.9999
10:PTX vs. 100:PLGA NPS_PTX-LOADED M08 AB-TARGETED	Yes	****	<0.0001
10:PTX vs. 100:PTX	Yes	*	0.0217
10:PTX vs. 500:PLGA NPS_EMPTY	No	ns	0.1927
10:PTX vs. 500:PLGA NPS_PTX-LOADED	Yes	****	<0.0001
10:PTX vs. 500:PLGA NPS_EMPTY M08 AB-TARGETED	Yes	*	0.0106
10:PTX vs. 500:PLGA NPS_PTX-LOADED M08 AB-TARGETED	Yes	****	<0.0001
10:PTX vs. 500:PTX	Yes	**	0.0067
50:PLGA NPS_EMPTY vs. 50:PLGA NPS_PTX-LOADED	Yes	**	0.001
50:PLGA NPS_EMPTY vs. 50:PLGA NPS_EMPTY M08 AB-TARGETED	No	ns	0.2239
50:PLGA NPS_EMPTY vs. 50:PLGA NPS_PTX-LOADED M08 AB-TARGETED	Yes	****	<0.0001
50:PLGA NPS_EMPTY vs. 50:PTX	No	ns	>0.9999
50:PLGA NPS_EMPTY vs. 100:PLGA NPS_EMPTY	No	ns	>0.9999
50:PLGA NPS_EMPTY vs. 100:PLGA NPS_PTX-LOADED	Yes	****	<0.0001
50:PLGA NPS_EMPTY vs. 100:PLGA NPS_EMPTY M08 AB-TARGETED	No	ns	>0.9999

50:PLGA NPS_EMPTY vs. 100:PLGA NPS_PTX-LOADED M08 AB-TARGETED	Yes	****	<0.0001
50:PLGA NPS_EMPTY vs. 100:PTX	Yes	*	0.0233
50:PLGA NPS_EMPTY vs. 500:PLGA NPS_EMPTY	No	ns	0.2041
50:PLGA NPS_EMPTY vs. 500:PLGA NPS_PTX-LOADED	Yes	****	<0.0001
50:PLGA NPS_EMPTY vs. 500:PLGA NPS_EMPTY M08 AB-TARGETED	Yes	*	0.0114
50:PLGA NPS_EMPTY vs. 500:PLGA NPS_PTX-LOADED M08 AB-TARGETED	Yes	****	<0.0001
50:PLGA NPS_EMPTY vs. 500:PTX	Yes	**	0.0072
50:PLGA NPS_PTX-LOADED vs. 50:PLGA NPS_EMPTY M08 AB-TARGETED	No	ns	0.4809
50:PLGA NPS_PTX-LOADED vs. 50:PLGA NPS_PTX-LOADED M08 AB-TARGETED	Yes	**	0.0065
50:PLGA NPS_PTX-LOADED vs. 50:PTX	Yes	**	0.0039
50:PLGA NPS_PTX-LOADED vs. 100:PLGA NPS_EMPTY	Yes	***	0.0006
50:PLGA NPS_PTX-LOADED vs. 100:PLGA NPS_PTX-LOADED	No	ns	0.9492
50:PLGA NPS_PTX-LOADED vs. 100:PLGA NPS_EMPTY M08 AB-TARGETED	Yes	**	0.0016
50:PLGA NPS_PTX-LOADED vs. 100:PLGA NPS_PTX-LOADED M08 AB-TARGETED	No	ns	0.0552
50:PLGA NPS_PTX-LOADED vs. 100:PTX	No	ns	0.9876
50:PLGA NPS_PTX-LOADED vs. 500:PLGA NPS_EMPTY	No	ns	0.5141
50:PLGA NPS_PTX-LOADED vs. 500:PLGA NPS_PTX-LOADED	No	ns	0.3092
50:PLGA NPS_PTX-LOADED vs. 500:PLGA NPS_EMPTY M08 AB-TARGETED	No	ns	0.9993
50:PLGA NPS_PTX-LOADED vs. 500:PLGA NPS_PTX-LOADED M08 AB-TARGETED	Yes	*	0.0189
50:PLGA NPS_PTX-LOADED vs. 500:PTX	No	ns	>0.9999
50:PLGA NPS_EMPTY M08 AB-TARGETED vs. 50:PLGA NPS_PTX-LOADED M08 AB-TARGETED	Yes	****	<0.0001
50:PLGA NPS_EMPTY M08 AB-TARGETED vs. 50:PTX	No	ns	0.5428
50:PLGA NPS_EMPTY M08 AB-TARGETED vs. 100:PLGA NPS_EMPTY	No	ns	0.1384
50:PLGA NPS_EMPTY M08 AB-TARGETED vs. 100:PLGA NPS_PTX-LOADED	Yes	*	0.0222
50:PLGA NPS_EMPTY M08 AB-TARGETED vs. 100:PLGA NPS_EMPTY M08 AB-TARGETED	No	ns	0.3156
50:PLGA NPS_EMPTY M08 AB-TARGETED vs. 100:PLGA NPS_PTX-LOADED M08 AB-TARGETED	Yes	***	0.0002
50:PLGA NPS_EMPTY M08 AB-TARGETED vs. 100:PTX	No	ns	0.9989
50:PLGA NPS_EMPTY M08 AB-TARGETED vs. 500:PLGA NPS_EMPTY	No	ns	>0.9999
50:PLGA NPS_EMPTY M08 AB-TARGETED vs. 500:PLGA NPS_PTX-LOADED	Yes	**	0.0016
50:PLGA NPS_EMPTY M08 AB-TARGETED vs. 500:PLGA NPS_EMPTY M08 AB-TARGETED	No	ns	0.9834
50:PLGA NPS_EMPTY M08 AB-TARGETED vs. 500:PLGA NPS_PTX-LOADED M08 AB-TARGETED	Yes	****	<0.0001
50:PLGA NPS_EMPTY M08 AB-TARGETED vs. 500:PTX	No	ns	0.9472
50:PLGA NPS_PTX-LOADED M08 AB-TARGETED vs. 50:PTX	Yes	****	<0.0001
50:PLGA NPS_PTX-LOADED M08 AB-TARGETED vs. 100:PLGA NPS_EMPTY	Yes	****	<0.0001
50:PLGA NPS_PTX-LOADED M08 AB-TARGETED vs. 100:PLGA NPS_PTX-LOADED	No	ns	0.2032
50:PLGA NPS_PTX-LOADED M08 AB-TARGETED vs. 100:PLGA NPS_EMPTY M08 AB-TARGETED	Yes	****	<0.0001

50:PLGA NPS_PTX-LOADED M08 AB-TARGETED vs. 100:PLGA NPS_PTX-LOADED M08 AB-TARGETED	No	ns	0.9998
50:PLGA NPS_PTX-LOADED M08 AB-TARGETED vs. 100:PTX	Yes	***	0.0003
50:PLGA NPS_PTX-LOADED M08 AB-TARGETED vs. 500:PLGA NPS_EMPTY	Yes	****	<0.0001
50:PLGA NPS_PTX-LOADED M08 AB-TARGETED vs. 500:PLGA NPS_PTX-LOADED	No	ns	0.8618
50:PLGA NPS_PTX-LOADED M08 AB-TARGETED vs. 500:PLGA NPS_EMPTY M08 AB-TARGETED	Yes	***	0.0006
50:PLGA NPS_PTX-LOADED M08 AB-TARGETED vs. 500:PLGA NPS_PTX-LOADED M08 AB-TARGETED	No	ns	>0.9999
50:PLGA NPS_PTX-LOADED M08 AB-TARGETED vs. 500:PTX	Yes	***	0.0009
50:PTX vs. 100:PLGA NPS_EMPTY	No	ns	>0.9999
50:PTX vs. 100:PLGA NPS_PTX-LOADED	Yes	***	0.0001
50:PTX vs. 100:PLGA NPS_EMPTY M08 AB-TARGETED	No	ns	>0.9999
50:PTX vs. 100:PLGA NPS_PTX-LOADED M08 AB-TARGETED	Yes	****	<0.0001
50:PTX vs. 100:PTX	No	ns	0.0823
50:PTX vs. 500:PLGA NPS_EMPTY	No	ns	0.509
50:PTX vs. 500:PLGA NPS_PTX-LOADED	Yes	****	<0.0001
50:PTX vs. 500:PLGA NPS_EMPTY M08 AB-TARGETED	Yes	*	0.0421
50:PTX vs. 500:PLGA NPS_PTX-LOADED M08 AB-TARGETED	Yes	****	<0.0001
50:PTX vs. 500:PTX	Yes	*	0.027
100:PLGA NPS_EMPTY vs. 100:PLGA NPS_PTX-LOADED	Yes	****	<0.0001
100:PLGA NPS_EMPTY vs. 100:PLGA NPS_EMPTY M08 AB-TARGETED	No	ns	>0.9999
100:PLGA NPS_EMPTY vs. 100:PLGA NPS_PTX-LOADED M08 AB-TARGETED	Yes	****	<0.0001
100:PLGA NPS_EMPTY vs. 100:PTX	Yes	*	0.013
100:PLGA NPS_EMPTY vs. 500:PLGA NPS_EMPTY	No	ns	0.1251
100:PLGA NPS_EMPTY vs. 500:PLGA NPS_PTX-LOADED	Yes	****	<0.0001
100:PLGA NPS_EMPTY vs. 500:PLGA NPS_EMPTY M08 AB-TARGETED	Yes	**	0.0063
100:PLGA NPS_EMPTY vs. 500:PLGA NPS_PTX-LOADED M08 AB-TARGETED	Yes	****	<0.0001
100:PLGA NPS_EMPTY vs. 500:PTX	Yes	**	0.004
100:PLGA NPS_PTX-LOADED vs. 100:PLGA NPS_EMPTY M08 AB-TARGETED	Yes	****	<0.0001
100:PLGA NPS_PTX-LOADED vs. 100:PLGA NPS_PTX-LOADED M08 AB-TARGETED	No	ns	0.7509
100:PLGA NPS_PTX-LOADED vs. 100:PTX	No	ns	0.2154
100:PLGA NPS_PTX-LOADED vs. 500:PLGA NPS_EMPTY	Yes	*	0.0249
100:PLGA NPS_PTX-LOADED vs. 500:PLGA NPS_PTX-LOADED	No	ns	0.9979
100:PLGA NPS_PTX-LOADED vs. 500:PLGA NPS_EMPTY M08 AB-TARGETED	No	ns	0.3642
100:PLGA NPS_PTX-LOADED vs. 500:PLGA NPS_PTX-LOADED M08 AB-TARGETED	No	ns	0.4359
100:PLGA NPS_PTX-LOADED vs. 500:PTX	No	ns	0.4857
100:PLGA NPS_EMPTY M08 AB-TARGETED vs. 100:PLGA NPS_PTX-LOADED M08 AB-TARGETED	Yes	****	<0.0001
100:PLGA NPS_EMPTY M08 AB-TARGETED vs. 100:PTX	Yes	*	0.0365
100:PLGA NPS_EMPTY M08 AB-TARGETED vs. 500:PLGA NPS_EMPTY	No	ns	0.2901

100:PLGA NPS_EMPTY M08 AB-TARGETED vs. 500:PLGA NPS_PTX-LOADED	Yes	****	<0.0001
100:PLGA NPS_EMPTY M08 AB-TARGETED vs. 500:PLGA NPS_EMPTY M08 AB-TARGETED	Yes	*	0.0181
100:PLGA NPS_EMPTY M08 AB-TARGETED vs. 500:PLGA NPS_PTX-LOADED M08 AB-TARGETED	Yes	****	<0.0001
100:PLGA NPS_EMPTY M08 AB-TARGETED vs. 500:PTX	Yes	*	0.0114
100:PLGA NPS_PTX-LOADED M08 AB-TARGETED vs. 100:PTX	Yes	**	0.0025
100:PLGA NPS_PTX-LOADED M08 AB-TARGETED vs. 500:PLGA NPS_EMPTY	Yes	***	0.0002
100:PLGA NPS_PTX-LOADED M08 AB-TARGETED vs. 500:PLGA NPS_PTX-LOADED	No	ns	>0.9999
100:PLGA NPS_PTX-LOADED M08 AB-TARGETED vs. 500:PLGA NPS_EMPTY M08 AB-TARGETED	Yes	**	0.0051
100:PLGA NPS_PTX-LOADED M08 AB-TARGETED vs. 500:PLGA NPS_PTX-LOADED M08 AB-TARGETED	No	ns	>0.9999
100:PLGA NPS_PTX-LOADED M08 AB-TARGETED vs. 500:PTX	Yes	**	0.0082
100:PTX vs. 500:PLGA NPS_EMPTY	No	ns	0.9994
100:PTX vs. 500:PLGA NPS_PTX-LOADED	Yes	*	0.0193
100:PTX vs. 500:PLGA NPS_EMPTY M08 AB-TARGETED	No	ns	>0.9999
100:PTX vs. 500:PLGA NPS_PTX-LOADED M08 AB-TARGETED	Yes	***	0.0008
100:PTX vs. 500:PTX	No	ns	>0.9999
500:PLGA NPS_EMPTY vs. 500:PLGA NPS_PTX-LOADED	Yes	**	0.0018
500:PLGA NPS_EMPTY vs. 500:PLGA NPS_EMPTY M08 AB-TARGETED	No	ns	0.9884
500:PLGA NPS_EMPTY vs. 500:PLGA NPS_PTX-LOADED M08 AB-TARGETED	Yes	****	<0.0001
500:PLGA NPS_EMPTY vs. 500:PTX	No	ns	0.9594
500:PLGA NPS_PTX-LOADED vs. 500:PLGA NPS_EMPTY M08 AB-TARGETED	Yes	*	0.0389
500:PLGA NPS_PTX-LOADED vs. 500:PLGA NPS_PTX-LOADED M08 AB-TARGETED	No	ns	0.9867
500:PLGA NPS_PTX-LOADED vs. 500:PTX	No	ns	0.0602
500:PLGA NPS_EMPTY M08 AB-TARGETED vs. 500:PLGA NPS_PTX-LOADED M08 AB-TARGETED	Yes	**	0.0017
500:PLGA NPS_EMPTY M08 AB-TARGETED vs. 500:PTX	No	ns	>0.9999
500:PLGA NPS_PTX-LOADED M08 AB-TARGETED vs. 500:PTX	Yes	**	0.0027

**After treatment**	**Two-way repeated measures ANOVA**			**with Tukey's post hoc test**	
Two-way RM ANOVA	Matching: Stacked				
Assume sphericity?	No				
Alpha	0.05				

Source of Variation	% of total variation	P value	P value summary	Significant?	Geisser-Greenhouse's epsilon
Time x treatment	8.904	<0.0001	****	Yes	
Time	55.76	<0.0001	****	Yes	1
treatment	22.79	0.0107	*	Yes	
Subject	9.697	<0.0001	****	Yes	
ANOVA table	SS	DF	MS	F (DFn, DFd)	P value
Time x treatment	222709	272	818.8	F (272, 680) = 7.824	P<0.0001
Time	1394625	68	20509	F (68.00, 680.0) = 196.0	P<0.0001
treatment	570109	4	142527	F (4, 10) = 5.877	P=0.0107
Subject	242522	10	24252	F (10, 680) = 231.7	P<0.0001
Residual	71162	680	104.7		
Data summary					
Number of columns (treatment)	5				
Number of rows (Time)	69				
Number of subjects (Subject)	15				
Number of missing values	0				

Repeated measures ANOVA, effect of treatment: (F4,10)=5.877; * P=0.0107, effect of time: (F68, 680)=196.0; **** P<0.0001, treatment x time interaction: (F272,680)= 7,824; **** P<0.0001

Post hoc analysis					
Compare column means (main column effect)					
Number of families	1				
Number of comparisons per family	10				
Alpha	0.05				
Tukey's multiple comparisons test	Mean Diff.	95.00% CI of diff.	Significant?	Summary	Adjusted P Value
PLGA NPS_EMPTY vs. PLGA NPS_PTX-LOADED M08 AB-TARGETED	-51.66	-65.05 to -38.27	Yes	****	<0.0001
PLGA NPS_EMPTY vs. PLGA NPS_EMPTY M08 AB-TARGETED	13.59	6.543 to 20.64	Yes	****	<0.0001
PLGA NPS_EMPTY vs. PLGA NPS_PTX-LOADED	-23.7	-34.36 to -13.04	Yes	****	<0.0001
PLGA NPS_EMPTY vs. FREE PTX	3.685	-5.961 to 13.33	No	ns	0.8331
PLGA NPS_PTX-LOADED M08 AB-TARGETED vs. PLGA NPS_EMPTY M08 AB-TARGETED	65.25	52.37 to 78.14	Yes	****	<0.0001

PLGA NPS_PTX-LOADED M08 AB-TARGETED vs. PLGA NPS_PTX-LOADED	27.96	12.83 to 43.10	Yes	****	<0.0001
PLGA NPS_PTX-LOADED M08 AB-TARGETED vs. FREE PTX	55.35	40.90 to 69.79	Yes	****	<0.0001
PLGA NPS_EMPTY M08 AB-TARGETED vs. PLGA NPS_PTX-LOADED	-37.29	-47.31 to -27.27	Yes	****	<0.0001
PLGA NPS_EMPTY M08 AB-TARGETED vs. FREE PTX	-9.908	-18.84 to -0.9801	Yes	*	0.0211
PLGA NPS_PTX-LOADED vs. FREE PTX	27.38	15.41 to 39.35	Yes	****	<0.0001
